# Exome-wide association study reveals novel psoriasis susceptibility locus at *TNFSF15* and rare protective alleles in genes contributing to type I IFN signalling

**DOI:** 10.1093/hmg/ddx328

**Published:** 2017-08-24

**Authors:** Nick Dand, Sören Mucha, Lam C Tsoi, Satveer K Mahil, Philip E Stuart, Andreas Arnold, Hansjörg Baurecht, A David Burden, Kristina Callis Duffin, Vinod Chandran, Charles J Curtis, Sayantan Das, David Ellinghaus, Eva Ellinghaus, Charlotta Enerback, Tõnu Esko, Dafna D Gladman, Christopher E M Griffiths, Johann E Gudjonsson, Per Hoffman, Georg Homuth, Ulrike Hüffmeier, Gerald G Krueger, Matthias Laudes, Sang Hyuck Lee, Wolfgang Lieb, Henry W Lim, Sabine Löhr, Ulrich Mrowietz, Martina Müller-Nurayid, Markus Nöthen, Annette Peters, Proton Rahman, André Reis, Nick J Reynolds, Elke Rodriguez, Carsten O Schmidt, Sarah L Spain, Konstantin Strauch, Trilokraj Tejasvi, John J Voorhees, Richard B Warren, Michael Weichenthal, Stephan Weidinger, Matthew Zawistowski, Rajan P Nair, Francesca Capon, Catherine H Smith, Richard C Trembath, Goncalo R Abecasis, James T Elder, Andre Franke, Michael A Simpson, Jonathan N Barker

**Affiliations:** 1 Division of Genetics and Molecular Medicine, Faculty of Life Sciences & Medicine, King's College London, London, UK; 2 Institute of Clinical Molecular Biology, Christian-Albrechts-University of Kiel, Kiel, Germany; 3 Department of Dermatology; 4 Department of Computational Medicine & Bioinformatics, University of Michigan Medical School, Ann Arbor, MI, USA; 5 Department of Biostatistics, School of Public Health, University of Michigan, Ann Arbor, MI, USA; 6 St John's Institute of Dermatology, Faculty of Life Sciences & Medicine, King's College London, London, UK; 7 Clinic and Polyclinic of Dermatology, University Medicine Greifswald, Greifswald, Germany; 8 Department of Dermatology, Venereology and Allergy, University Hospital Schleswig-Holstein, Campus Kiel, Kiel, Germany; 9 Institute of Infection, Inflammation and Immunity, University of Glasgow, Glasgow, UK; 10 Department of Dermatology, University of Utah, Salt Lake City, UT, USA; 11 Department of Medicine; 12 Department of Laboratory Medicine and Pathobiology; 13 Institute of Medical Science, University of Toronto, Toronto, ON, Canada; 14 Krembil Research Institute, University Health Network, Toronto, ON, Canada; 15 NIHR Biomedical Research Centre at South London and Maudsley NHS Foundation Trust and King’s College London, London, UK; 16 Social Genetic and Developmental Psychiatry Centre, Institute of Psychiatry, Psychology and Neuroscience, King’s College London, London, UK; 17 Division of Cell Biology and Dermatology, Department of Clinical and Experimental Medicine, Linköping University, Linköping, Sweden; 18 Estonian Biobank, Estonian Genome Center, University of Tartu, Tartu, Estonia; 19 Dermatology Centre, Salford Royal Hospital, Manchester Academic Health Science Centre, University of Manchester, Manchester, UK; 20 Genomics Research Group, Department of Biomedicine, University of Basel, Basel, Switzerland; 21 Institute of Human Genetics, University of Bonn, Bonn, Germany; 22 Department of Functional Genomics, Interfaculty Institute for Genetics and Functional Genomics, University Medicine and Ernst-Moritz-Arndt-University Greifswald, Greifswald, Germany; 23 Institute of Human Genetics, Friedrich-Alexander-Universität Erlangen-Nürnberg, Erlangen, Germany; 24 I. Department of Medicine; 25 Institute of Epidemiology and Biobank PopGen, Christian-Albrechts-University of Kiel, Kiel, Germany; 26 Department of Dermatology, Henry Ford Hospital, Detroit, MI, USA; 27 Institute of Genetic Epidemiology, Helmholtz Zentrum Munich, Neuherberg, Germany; 28 Memorial University of Newfoundland, St. John's, NL, Canada; 29 Dermatological Sciences, Institute of Cellular Medicine, Newcastle University Medical School, Newcastle upon Tyne, UK; 30 Department of Dermatology, Royal Victoria Infirmary, Newcastle Hospitals NHS Foundation Trust, Newcastle upon Tyne, UK; 31 Institute for Community Medicine, Study of Health in Pomerania/KEF, University Medicine Greifswald, Greifswald, Germany; 32 Dermatology Centre, Salford Road NHS Foundation Trust, Manchester Academic Health Science Centre, The University of Manchester, Manchester, UK; 33 Department of Dermatology, University Medical Center Schleswig-Holstein, Campus Kiel, Kiel, Germany; 34 Ann Arbor Veterans Hospital, Ann Arbor, MI, USA

## Abstract

Psoriasis is a common inflammatory skin disorder for which multiple genetic susceptibility loci have been identified, but few resolved to specific functional variants. In this study, we sought to identify common and rare psoriasis-associated gene-centric variation. Using exome arrays we genotyped four independent cohorts, totalling 11 861 psoriasis cases and 28 610 controls, aggregating the dataset through statistical meta-analysis. Single variant analysis detected a previously unreported risk locus at *TNFSF15* (rs6478108; *P* = 1.50 × 10^−8^, OR = 1.10), and association of common protein-altering variants at 11 loci previously implicated in psoriasis susceptibility. We validate previous reports of protective low-frequency protein-altering variants within *IFIH1* (encoding an innate antiviral receptor) and *TYK2* (encoding a Janus kinase), in each case establishing a further series of protective rare variants (minor allele frequency < 0.01) via gene-wide aggregation testing (*IFIH1*: *p*_burden_ = 2.53 × 10^−7^, OR = 0.707; *TYK2*: *p*_burden_ = 6.17 × 10^−4^, OR = 0.744). Both genes play significant roles in type I interferon (IFN) production and signalling. Several of the protective rare and low-frequency variants in *IFIH1* and *TYK2* disrupt conserved protein domains, highlighting potential mechanisms through which their effect may be exerted.

## Introduction

Psoriasis is a common inflammatory hyperproliferative skin disorder with a significant genetic component to disease pathogenesis (1–3). It affects up to 2% of people worldwide, with affected individuals suffering high social and economic costs and increased morbidity and mortality (1,4). Previous large-scale genome-wide association studies and meta-analyses have identified 63 loci that contribute to psoriasis susceptibility in populations of European origin (5–16). Recent studies have refined the understanding of the allelic architecture of psoriasis risk at several of these loci, including the major histocompatibility complex (MHC), through the detection of multiple independent secondary signals (8,12,17,18). Many psoriasis risk loci harbour genes encoding components of disease-relevant biological processes, including innate and adaptive immune pathways and skin barrier function (2). Nevertheless, the precise molecular mechanisms through which the associated genetic variation confers psoriasis susceptibility remain uncertain for the majority of these signals (19). The identification of disease-associated protein-altering variation, including the effects of rare alleles, has the potential to illuminate the mechanisms underpinning the pathogenic process and to identify putative targets for therapeutic intervention. Notably in psoriasis, investigation of the protective common allele encoding a glutamine residue at position 381 of the interleukin-23 (IL-23) receptor has validated aberrant Th17 signalling as a key disease driver (20), consistent with the remarkable efficacy of therapeutics targeting this pathway (21).

Potential roles for common and low-frequency protein-altering variants in psoriasis susceptibility have been investigated in the Han Chinese (22,23) and European (8) populations, but until now the contribution of rare protein-altering alleles to the disease architecture has not been systematically explored in any population. Here, we present the most comprehensive investigation to date of protein-altering variation in psoriasis risk in the European population. The analysis encompasses four independent exome array association studies, referred to here as the UK, Estonia, Germany and Michigan studies. These were combined through meta-analysis to total 11 861 psoriasis cases and 28 610 controls (Supplementary Material, Table S1). Our analysis focused on genetic variation outside of the MHC region, in which the psoriasis-associated HLA-C*06:02 allele and independent secondary signals have been the subject of extensive investigation elsewhere (17,18,24–26). After quality control (QC), 167 587 single nucleotide variants (SNVs) that were successfully genotyped in each of the four cohorts were investigated. This set included 155 870 variants located within protein coding regions (and associated splice sites) of the genome and a further 11 717 non-coding SNVs including many tagging previously reported disease-associated SNVs. The allele frequency spectrum of the set of genotyped variants is skewed towards rare and low-frequency variants (Supplementary Material, Table S3).

## Results

Single marker association testing was performed in each of the four case-control cohorts using a linear mixed model with an empirically estimated relatedness matrix to control for population structure (27), and results were aggregated across studies via meta-analysis (Materials and Methods). Results for all variants achieving an association *P*-value *P* < 1 × 10^−5^ are summarised in Supplementary Material, Table S5.

### Single marker association tests uphold established psoriasis susceptibility loci

Of 67 previously reported independent psoriasis susceptibility signals across 63 loci, we were able to test for disease association at 24, either directly using the reported lead SNV or via a proxy (*r*^2^ > 0.8 with the reported lead SNV). We observe significant disease associations at 23 (20 with genome-wide significance, *P* < 5 × 10^−8^, and three with *P* < 10^−4^; Supplementary Material, Table S6). We find no evidence of association at the recently reported 13q14.11 locus that encompasses *COG6* (10) (rs7993214: *P* = 0.0589; OR = 1.04). It should be noted that before QC the UK, Estonia, Germany and Michigan studies collectively included 7885 psoriasis cases that were present in previously published analyses (6,8,12,14) (Supplementary Material, Table S1). As such, the associations that originate from these previous reports should not be considered independently replicated here.

### Previously unreported genome-wide association at one locus

We detect genome-wide significant association at one further locus, mapping to *TNFSF15* at 9q32. An association is observed with the intronic variant rs6478108 (*P* = 1.50 × 10^−8^; OR = 1.10; Supplementary Material, Table S5). *TNFSF15*, encoding a member of the tumor necrosis factor superfamily of cytokines, is primarily expressed in endothelial cells and has previously been implicated in susceptibility to Crohn’s disease (28). Although a previous study found nominal association between variants in this locus and psoriasis susceptibility in a Hungarian population (29), we establish 9q32 as a genome-wide significant susceptibility locus for the first time here.

### Established association signals map to protein-altering variants at 11 susceptibility loci

Within psoriasis susceptibility loci, an ongoing challenge is to fine-map association signals to determine the underlying causal variants. Disease-associated protein-altering variants represent plausible candidates through which psoriasis risk is conferred. We searched for protein-altering SNVs with a consistent direction of effect across all four studies and exome-wide significant association (*P* ≤ 3.0 × 10^−7^). We found 19 such variants within 11 different loci, all of which were previously reported susceptibility loci (Table 1; Supplementary Material, Table S5). These observations extend the list of putative causal protein-altering alleles previously reported (8), most notably defining an additional candidate causal variant in *ERAP1* (rs30187: p.K528R; *P* = 2.19 × 10^−11^) that is predicted to be damaging by PolyPhen-2 and has a CADD score of 20.6 (Supplementary Material, Table S5). Furthermore, conditional analysis indicates that this variant can account for the observed association of rs27432, the lead SNV in this locus reported by Tsoi *et al.* (8) (Supplementary Material, Table S5).

**Table 1.** Exome-wide significant protein-altering variants

Locus	Protein- altering SNV	hg19 position	Ref/Alt	Gene	Consequence	Variant effect prediction	AAF_case_	AAF_cont_	OR	Meta-analysis *P*-value	Corresponding established signal
									(95% CI)		
1p31.3	rs11209026	67705958	G/A	*IL23R*	R381Q	P, C	0.046	0.062	0.719	2.00 × 10^−18^	rs9988642
									(0.667–0.774)		
1q21.3	rs1332500	152692074	G/C	*C1orf68*	S26T		0.376	0.348	1.123	1.07 × 10^−12^	rs6677595
									(1.086–1.161)		
	rs873775	152692472	A/C	*C1orf68*	T159P		0.376	0.348	1.123	1.11 × 10^−12^	rs6677595
									(1.086–1.161)		
2q24.2	rs1990760	163124051	C/T	*IFIH1*	A946T		0.647	0.618	1.161	4.73 × 10^−18^	rs1990760
									(1.122–1.200)		
	rs35667974	163124637	T/C	*IFIH1*	I923V	P	0.010	0.020	0.548	1.10 × 10^−15^	rs1990760
									(0.473–0.634)		
5q15	rs27044	96118852	G/C	*ERAP1*	Q730E		0.698	0.725	0.869	1.28 × 10^−13^	rs27432
									(0.839–0.900)		
	rs30187	96124330	T/C	*ERAP1*	K528R	P, C	0.633	0.663	0.886	2.19 × 10^−11^	rs27432
									(0.857–0.916)		
5q31.1	rs20541	131995964	A/G	*IL13*	Q144R		0.814	0.783	1.170	3.59 × 10^−13^	rs20541
									(1.123–1.219)		
6q21	rs33980500	111913262	C/T	*TRAF3IP2*	D19N	S, P, C	0.108	0.075	1.451	1.92 × 10^−39^	rs33980500
									(1.374–1.533)		
	rs13190932	111913070	G/A	*TRAF3IP2*	R83W	S	0.084	0.060	1.404	1.34 × 10^−28^	rs33980500
									(1.320–1.492)		
	rs458017	111696091	T/C	*REV3L*	Y1156C		0.085	0.064	1.313	1.37 × 10^−19^	rs33980500
									(1.236–1.395)		
12q13.3	rs2066807	56740682	C/G	*STAT2*	M594I		0.053	0.073	0.729	1.56 × 10^−17^	rs2066808
									(0.681–0.781)		
16p11.2	rs9938550	30999142	A/G	*HSD3B7*	T250A		0.599	0.626	0.882	4.74 × 10^−13^	rs10782001
									(0.853–0.911)		
19p13.2	rs34536443	10463118	G/C	*TYK2*	P1104A	S, P, C	0.023	0.044	0.506	1.72 × 10^−42^	rs34536443
									(0.458–0.558)		
	rs2304256	10475652	C/A	*TYK2*	V362F		0.243	0.280	0.814	2.88 × 10^−23^	rs34536443 and rs12720356
									(0.784–0.844)		
	rs12720356	10469975	A/C	*TYK2*	I684S	S, P	0.068	0.083	0.763	1.39 × 10^−16^	rs12720356
									(0.716–0.812)		
	rs1051738	10577843	C/A	*PDE4A*	A736E		0.166	0.184	0.879	2.02 × 10^−7^	rs34536443
									(0.843–0.918)		
19q13.33	rs602662	49206985	G/A	*FUT2*	G258S	P	0.511	0.467	1.090	3.29 × 10^−8^	rs281379
									(1.056–1.126)		
20q13.13	rs4647958	48600631	T/C	*SNAI1*	V118A		0.141	0.125	1.161	9.21 × 10^−10^	rs495337
									(1.107–1.217)		

AAF = alternative allele frequency; OR = estimated odds ratio; variant effect prediction: S = predicted “Damaging” by SIFT; *P =* predicted “probably damaging” by PolyPhen-2; C = CADD score > 20.

### Rare variant aggregation tests identify protective alleles for type I IFN genes

Despite the substantial sample size of the current study, evaluation of the contribution of individual rare and low-frequency variants to psoriasis susceptibility is limited by statistical power to detect association. We therefore performed a series of gene-based tests, aggregating variants with low minor allele frequency (MAF). At each MAF threshold (0.01 or 0.05) we performed a burden test to detect an excess of rare alleles in cases or controls, and a SKAT test, which is designed to detect scenarios in which the effects of the aggregated variants have different direction or magnitude (30) (Materials and Methods). This testing regime identified two genes, *IFIH1* and *TYK2*, with exome-wide significant evidence of association (*p*_gene_ < 2.5 × 10^−6^; Table 2). Both *IFIH1* and *TYK2* are located in loci previously implicated in common variant studies of psoriasis risk (5).

**Table 2.** Exome-wide significant gene-based associations

Locus	Gene	SNVs conditioned on	MAF < 0.01					MAF < 0.05				
			*p*_burden_	OR (95% CI)	*p*_SKAT_	*n*_SNVs_	cMAF	*p*_burden_	OR (95% CI)	*p*_SKAT_	*n*_SNVs_	cMAF
***Unconditioned analysis***												
2q24.2	*IFIH1*	-	**2.53 × 10^−7^**	0.707	6.02 × 10^**−**^^5^	24	0.0261	**1.84 × 10^−19^**	0.620	**1.19 × 10^−20^**	25	0.0461
				(0.626–0.799)					(0.564–0.682)
19p13.2	*TYK2*	-	6.17 × 10^**−**^^4^	0.744	2.82 × 10^**−**^^4^	17	0.0115	**1.47 × 10^−39^**	0.593	**6.34 × 10^−41^**	19	0.0675
				(0.626–0.885)					(0.549–0.641)
***Conditional analysis***												
2q24.2	*IFIH1*	rs35667974 and rs1990760	**1.36 × 10^−8^**	0.687	4.46 × 10^**−**^^6^	24	0.0261	**1.36 × 10^−8^**	0.687	4.46 × 10^**−**^^6^	24	0.0261
				(0.607–0.776)					(0.607–0.776)
19p13.2	*TYK2*	rs34536443, rs2304256 and rs12720356	1.45 × 10^**−**^^4^	0.728	7.21 × 10^**−**^^5^	17	0.0115	1.85 × 10^**−**^^5^	0.790	6.53 × 10^**−**^^5^	18	0.0239
				(0.611–0.868)					(0.701–0.890)


MAF = minor allele frequency; OR = odds ratio estimated by collapsing test; *n*_SNVs_ = number of SNVs included in test (this may vary between unconditioned and conditional analysis since SNVs to be conditioned on which are sufficiently rare are not included in the test statistic for the conditional test); cMAF = cumulative minor allele frequency of SNVs included in test. Exome-wide significant *P*-values (*p*_gene_ < 2.5 × 10^−6^) are indicated in bold.

**Figure 1.**
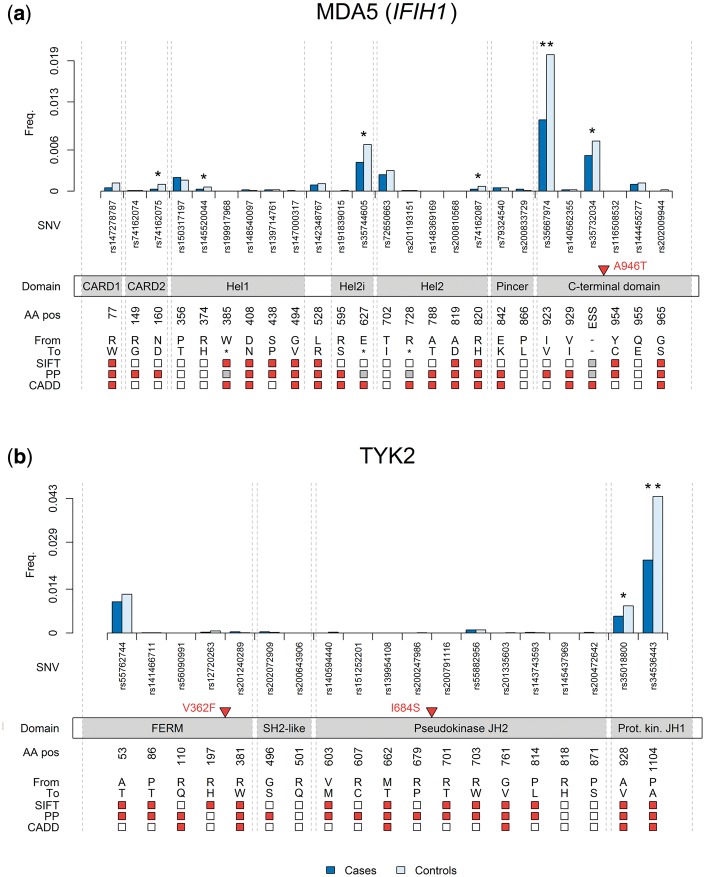
Rare and low-frequency protein-altering variants in IFIH1 and TYK2. Frequency of alternative allele in cases and controls across all four studies for rare and low-frequency variants, displayed by protein consequence. ** designates exome-wide significant association (*P* < 3.0 × 10^−7^); * designates nominally significant association (*P* < 0.05). Common protein-altering variants that we report to be associated are marked by red triangles. Variant effect predictions (by SIFT, PolyPhen-2 and CADD) are red where a substitution is predicted to be damaging, white where it is not, and grey where no prediction was possible. SNV = single nucleotide variant; ESS = essential splice site; AA pos = amino acid position; PP = PolyPhen-2. (**A**) *IFIH1* variants (MDA5 protein): CARD = caspase activation recruitment domain; Hel = helicase domain. (**B**) *TYK2* variants: FERM = 4.1/ezrin/radixin/moesin domain; SH2-like = Src homology 2-like domain; JH2 = JAK-homology 2; Prot. kin. JH1 = protein kinase JAK-homology 1.

In *IFIH1*, single marker association testing had identified two genome-wide significant psoriasis-associated protein-coding variants: the common rs1990760 (p.A946T; MAF_controls_ = 0.38; *P* = 4.73 × 10^−18^; OR = 0.86) and the low-frequency rs35667974 (p.I923V; MAF_controls_ = 0.02; *P* = 1.10 × 10^−15^; OR = 0.55). The latter contributes to the observed gene-based association of variants in *IFIH1* with MAF < 0.05 (*p*_SKAT_ = 1.19 × 10^−20^; *p*_burden_ = 1.84 × 10^−19^), although we also observe evidence of association with a MAF threshold of 0.01 (*p*_burden_ = 2.53 × 10^−7^; *p*_SKAT_ = 6.02 × 10^−5^). This association remains when conditioning on both rs35667974 and rs1990760 (*p*_conditional-burden_ = 1.36 × 10^−8^; *p*_conditional-SKAT_ = 4.46 × 10^−6^), or on either individually (Supplementary Material, Table S8). Examination of allele frequencies of individual rare and low-frequency coding SNVs in *IFIH1* (Supplementary Material, Table S10) reveals differences between cases and controls (*P* < 0.05) for six variants, each located within predicted functional domains of MDA5, the antiviral receptor encoded by this gene (Fig. 1A). Notably, at each of these sites the minor allele is associated with decreased psoriasis risk, consistent with a previous study reporting two rare variant associations in this gene (31).

In *TYK2*, which encodes one of the Janus family of kinases (32), we detect a gene-level association across variants with MAF < 0.05 (*p*_SKAT_ = 6.34 × 10^−41^; *p*_burden_ = 1.47 × 10^−39^). As with *IFIH1*, a strong single-variant association contributes to the aggregated signal (rs34536443: MAF_cases_ = 0.023; MAF_controls_ = 0.044; *P* = 1.72 × 10^−42^; OR = 0.51). Nevertheless, the variants with MAF < 0.01 also display evidence for disease association (*p*_SKAT_ = 2.82 × 10^−4^; *p*_burden_ = 6.17 × 10^−4^). There is a complex linkage disequilibrium (LD) structure between individual variants that have been previously reported at this locus (see Discussion). Our data suggest that the observed aggregate rare variant association is independent of the single marker associations (*p*_conditional-SKAT_ = 7.21 × 10^−5^; *p*_conditional-burden_ = 1.45 × 10^−4^), although a suitable proxy to facilitate conditional analysis with the common disease-associated intronic SNV rs280519 was unavailable for this analysis (5). The low-frequency variant rs34536443 results in a substitution in TYK2’s kinase domain (p.P1104A), as does the only rare variant that is nominally associated with a single marker test (rs35018800: *P* = 0.0003; OR = 0.68; Fig. 1B;Supplementary Material, Table S10).

Since *IFIH1* and *TYK2* are located in known psoriasis susceptibility loci, we further scrutinized genes in all previously reported psoriasis susceptibility loci (Online Methods). We observed suggestive evidence for aggregated rare variant association at four further genes (*IL23R*, *TNFAIP3*, *DDX58* and *STAT2*; Supplementary Material, Table S9), the rare alleles displaying a protective effect in each case. Of these, we note that *DDX58* (*p*_burden_ = 3.01 × 10^−5^; *p*_SKAT_ = 7.82 × 10^−5^ for MAF < 0.05) encodes RIG-I, a paralog of the MDA5 receptor (encoded by *IFIH1*) with a closely related function (33). The most strongly associated single marker in the region (rs657454; *P* = 2.16 × 10^−5^; OR = 1.08; MAF_controls_ = 0.38) is not responsible for the observed association (*p*_conditional-burden_ = 3.15 × 10^−5^; *p*_conditional-SKAT_ = 3.07 × 10^−5^), although without a suitable proxy for conditional analysis we cannot fully rule out that the association is driven by the previously reported (8) common SNV rs11795343 (*r*^2^ with rs657454 = 0.411). Furthermore *STAT2* (*p*_burden_ = 3.80 × 10^−5^; *p*_SKAT_ = 9.48 × 10^−5^ for MAF < 0.05), like *IFIH1, DDX58* and *TYK2*, also encodes an important component of the type I IFN signalling pathway.

## Discussion

The systematic analysis of protein-altering variation reported here allows a thorough examination of the contribution of functional genetic mechanisms to psoriasis risk. For each locus in which we identified robustly associated single variants, the Supplementary Note provides a summary of evidence for functional involvement. For several loci (including those harbouring *IL23R*, *IL13* and *STAT2*), the most strongly associated functional variants remain those previously suggested by Tsoi *et al.* (8). Findings at other loci (1q21.3, 6q21, 16p11.2, 20q13.13) offered less clear interpretation but we did not find sufficient evidence to reject existing disease models involving candidate disease genes *LCE3B/C*, *TRAF3IP2*, *FBXL19* and *RNF114*, respectively (7,34–36). We note the significant association of rs30187 in *ERAP1* (*P* = 2.19 × 10^−11^; OR = 0.89), a missense variant that can explain the association signal at the previously proposed causal variant rs27044 (8) and which, unlike the latter SNV, is predicted to be deleterious by both PolyPhen-2 and CADD (Supplementary Material, Table S5). We also identified one significantly associated missense variant in the recently reported 19q13.33 locus (16): rs602662 in the gene *FUT2* (*P* = 3.29 × 10^−8^; OR = 1.09). This gene encodes α-(1,2) fucosyltransferase, a Lewis antigen system enzyme that is central to determining an individual’s secretor status (37) and is associated with protection from and susceptibility to certain viral, bacterial and fungal infections (38–40).

TYK2, as a Janus kinase, is widely expressed and facilitates a broad range of intracellular-signalling processes (32). It provides another link between psoriasis-associated innate and adaptive immune pathways, having been shown, for example, both to mediate Th17 cell responses to IL-23 signalling and Th1 responses to IL-12 signalling, and to regulate type I interferon (IFN) signalling (41,42). Our results highlight the complex LD structure at this locus, with protective associations at two previously identified independent missense variants which are each predicted to impair protein function (rs34536443 described earlier; rs12720356: *P* = 1.39 × 10^−16^; OR = 0.76; MAF_cases_ = 0.068; MAF_controls_ = 0.083) (5,8). A third SNV, rs2304256 (*P* = 2.88 × 10^−23^; OR = 0.81; MAF_cases_ = 0.243; MAF_controls_ = 0.280), is in weak LD with both rs34536443 and rs12720356 (*r*^2^ = 0.107 and 0.290, respectively) and its association disappears when conditioning on either SNV (*p*_conditional_ = 0.0559 and *p*_conditional_ = 0.1063, respectively). Our data included no proxy SNV for rs280519, another independent psoriasis signal with which rs2304256 is also in moderate LD (*r*^2^ = 0.357). In other immune-mediated diseases, rs2304256 has been shown to represent a synthetic association due to neighbouring rarer variants including rs34536443 and rs12720356 (43,44). It is notable that the observed association at *TYK2* under the rare variant aggregation tests is driven by two protective alleles which disrupt TYK2’s kinase domain (Fig. 1B). This may suggest that the catalytic activity of TYK2 helps to initiate and maintain the positive feedback loops that culminate in psoriatic inflammation. Indeed, our independently associated common SNV rs12720356 leads to the substitution p.I684S within the pseudokinase JAK-homology 2 domain (32,42,45), while conversely our likely synthetic association rs2304256 (p.V362F) impacts neither kinase domain.

*IFIH1* encodes the innate antiviral receptor MDA5, which detects and binds to double-stranded RNA, promoting a pro-inflammatory type I interferon (IFN) response (46). Rare variants in *IFIH1*, including three of the six variants underlying our aggregation tests that exhibit nominal disease association, have previously been shown to be protective for type I diabetes (47), with evidence that the associated rare alleles lead to a decrease in downstream IFNβ expression arising from impaired signal propagation (48). It is evident that this pathway is also relevant to psoriasis pathogenesis indicating potential shared mechanisms at this locus in these immune-mediated diseases. The most strongly associated rare variant is rs35667974, whose minor (C) allele exhibits a large protective effect (*P* = 1.10 × 10^−15^; MAF_cases_ = 0.010; MAF_controls_ = 0.020; OR = 0.55). This SNV is one of two independent rare variants at the *IFIH1* locus previously implicated by Li *et al.* in psoriasis susceptibility (31), the other (rs10930046) having not been tested in our study. Our single marker tests identified one further protein-altering variant with exome-wide significant disease association, but the association at the common variant rs1990760 (reported previously (10)) is lost when conditioning on rs35667974 (*p*_conditional_ = 0.9619), implying that it is a consequence of LD. It is also not predicted to be damaging (Supplementary Material, Table S5), which suggests rs35667974 could represent the more likely functional variant at this locus.

Several of the variants we report here exhibit some degree of effect size heterogeneity in our meta-analysis (Supplementary Material, Table S5). Notably, many variants display only modest evidence for association in the Estonia cohort, likely driven by the relatively small sample size for this study (Supplementary Material, Table S1). However, each of the variants has consistent direction of effect across all four studies and are consistent with established psoriasis susceptibility signals, and as such represent robust associations.

The results of our meta-analysis contribute to our understanding of several mechanisms of psoriasis pathogenesis. However, it might have been anticipated that the large study size and exome-wide genotyping coverage would result in more novel biological insights than was borne out in practice. We therefore examined how completely the 167 587 variants in our study covered the autosomal protein-altering variants (outside the MHC region and predicted to impair protein function) that are observed in 33 370 European whole-exome- or whole-genome-sequenced samples in the ExAC reference dataset (Supplementary Material, Table S11). Of 14 123 common variants (MAF ≥ 0.05) in ExAC, 5487 (38.9%) are absent from at least one version of the exome arrays used across our four studies. A further 1564 (11.1%) were removed from the analysis during genotyping QC, meaning that 7072 (50.1%) were tested in our analysis. A similar proportion of low-frequency (0.01 ≤ MAF < 0.05) and rare (0.001 ≤ MAF < 0.01) SNVs were tested. As expected, coverage of very rare variants with MAF below 0.001 was substantially sparser, the drop in coverage being more pronounced the lower the MAF (Supplementary Material, Table S11).

To assess the impact of this incomplete coverage on our ability to map established psoriasis susceptibility signals to functional variants, we searched for all SNVs that are in moderate LD with a previously reported association (*r*^2^ > 0.2) in 1000 Genomes European samples and predicted to impair protein function by at least one of SIFT, PolyPhen-2 and CADD (Supplementary Material, Table S12). We found 23 such variants, of which 11 (47.8%, consistent with overall coverage) were not tested in our meta-analysis and are therefore potentially interesting candidate variants for future association testing. The 12 variants which were tested include 8 with strong evidence of association (and present in Table 1). The remaining four variants are not exome-wide significantly associated with psoriasis susceptibility, but none are in strong LD with the corresponding established signal (*r*^2^ range 0.29-0.69; Supplementary Material, Table S12).

We note that the rare and low-frequency variants found to be associated in this study display broadly protective effects on psoriasis risk. We cannot exclude that this is due to selection bias, since the exome array design is based largely on variants observed in whole exome sequencing studies of a range of complex traits, which do not include psoriasis (http://genome.sph.umich.edu/wiki/Exome_Chip_Design). This could limit the probability that the array includes rare variants associated with increased psoriasis risk, either individually or via gene-wide aggregation tests.

Previously established risk loci account for around 28% of the estimated heritability of psoriasis (16). Based on the method of So *et al.* (49) we find that the newly reported association at *TNFSF15* explains 0.23% of estimated heritability (50). Aggregated rare and low-frequency variants (MAF < 0.05) in *IFIH1* account for 0.47% of estimated heritability (0.17% after conditioning on previously reported associations); for *TYK2* we estimate 0.80% (0.06% after conditional analysis). While these figures do not substantially increase the cumulative proportion of heritability explained to date, they do highlight the possibility that some fraction of the residual unexplained heritability will be due both to many as yet unidentified psoriasis susceptibility loci and to rare variants at existing loci. Further efforts to isolate such variants will require larger sample sizes and more comprehensive coverage of the full frequency spectrum of genetic variation.

In summary, we establish genome-wide significant psoriasis associations at the *TNFSF15* locus and identify a series of alleles at established psoriasis loci with plausible evidence for causality based on predicted effects on protein structure and function. Our investigation of alleles at the low end of the frequency spectrum with variant aggregation tests has expanded our understanding of the allelic architecture of psoriasis risk at the *IFIH1* and *TYK2* loci. The observation that rare alleles that disrupt conserved domains within each gene have protective effects is compatible with the hypothesis that the common ancestral alleles of *IFIH1* and *TYK2* contribute to a robust immune response to pathogens, but this comes at the expense of increased risk of immune-mediated disease. Our findings support a central role for type I IFN signalling in psoriasis pathogenesis, consistent with clinical observations that type I IFN therapy can induce or exacerbate psoriasis symptoms (51,52). They also highlight putative therapeutic mechanisms; the efficacy of other janus kinase inhibitors (53–55) suggest that TYK2 in particular may be a fruitful drug target.

## Materials and Methods

### Study samples and genotyping

The meta-analysis includes four independent studies, referred to as the UK, Estonia, Germany and Michigan studies. In each study, all samples were collected from unrelated individuals of European ancestry after obtaining written informed consent. Enrolment of subjects in each study was approved by the ethics boards of the participating institutions, in accordance with Declaration of Helsinki principles. All cases had been diagnosed with psoriasis vulgaris by a dermatologist. DNA was isolated from blood using standard methods.

#### UK data

Psoriasis cases (*n* = 1971) were recruited as previously described (8). Further cases (*n* = 960) were recruited from centres in the UK via the Biomarkers of Systemic Treatment Outcomes in Psoriasis (BSTOP) cohort study (www.kcl.ac.uk/lsm/research/divisions/gmm/departments/dermatology/Research/stru/groups/bstop/index.aspx; date last accessed August 15, 2017) after research ethics approval (REC reference 11/H0802/7). Unselected population-based controls (*n* = 6400) were obtained from the 1958 British Birth Cohort. Genotyping was performed using Illumina HumanExome-12 v1.1 BeadChip and Illumina HumanOmniExpressExome-8 v1.2 BeadChip for psoriasis cases, and Illumina HumanExome-12 v1.0 BeadChip for controls (Supplementary Material, Table S1).

#### German data

All German psoriasis cases (*n* = 2928) were recruited through local outpatient services at either the Department of Dermatology at Christian-Albrechts-University Kiel, or the Department of Dermatology and Allergy at the Technical University of Munich. The psoriasis cases were genotyped using Illumina HumanExome-12 v1.1, HumanCoreExome-12 v1.1B or HumanCoreExome-24 v1.0 A BeadChips. German healthy control individuals (*n* = 15 966) were obtained from the PopGen biobank, the KORA S4 survey (an independent population-based sample from the general population living in the region of Augsburg, southern Germany), the Heinz-Nixdorf Recall (HNR) cohort, Bonn, and the SHIP and SHIP-TREND cohorts (56) (from the Study of Health in Pomerania, a prospective longitudinal population-based cohort study in West Pomerania). German controls were genotyped using Illumina HumanExome-12 v1.0, HumanCoreExome-24 v1.0 A or HumanOmniExpressExome-8 v1.2 A BeadChips (Supplementary Material, Table S1).

#### Estonian data

All Estonian samples were provided by the population-based biobank of the Estonian Genome Center, University of Tartu. Subjects were recruited by general practitioners (GP) and physicians in the hospitals. Participants in the hospitals were randomly selected from individuals visiting GP offices or hospitals. Diagnosis of psoriasis on the basis of clinical symptoms was posed by a general practitioner and confirmed by a dermatologist (*n* = 1459). At the time of recruitment, the controls (*n* = 3167) did not report diagnosis of osteoarthritis, psoriasis, or autoimmune diseases. All Estonian samples were genotyped using Illumina HumanExome-12 v1.1 or HumanCoreExome-24 v1.0 BeadChips (Supplementary Material, Table S1).

#### Michigan data

Psoriasis cases (*n* = 6344) and unrelated, unaffected controls (*n* = 6085) of European Caucasian descent were collected in North America and Sweden (Supplementary Material, Table S1). The cohort was genotyped using the Affymetrix Axiom Biobank Plus Genotyping Array at the Affymetrix facility (Santa Clara, CA). In addition to the exome array content analysed in the present study, the chip included genome-wide and customized content analysed as part of a concurrent GWAS meta-analysis (16).

### Genotype calling and quality control

Initial genotype calling and QC was performed separately for each of the four studies. Subsequently a joint QC procedure was undertaken to ensure that consistent QC standards were adhered to (Supplementary Material, Table S2).

#### UK data

Genotype calling was performed separately for the three different chips using Illumina’s GenomeStudio Data Analysis software (samples clustered using GenTrain 2.0 algorithm). Sample QC was performed using PLINK (v1.07) (57) and R (58), with samples excluded based on call rate (< 0.95), suspected non-European ancestry, heterozygosity (±4 s.d. from the mean), array signal intensity (> 4 s.d. from the mean) and relatedness. SNVs were excluded due to call rate (< 0.99), deviations from Hardy-Weinberg equilibrium (*P* < 0.0001) and low GenomeStudio cluster separation score (< 0.4). We also excluded duplicate assays, tri-allelic variants and insertions/deletions from further analysis. zCall software (version 3) (59) was employed to improve genotype calling for samples and SNVs that passed the initial QC. Subsequently we excluded SNVs and samples having a revised call rate below 99% to give a total of 234 976 SNVs in 2431 cases and 5892 controls. Genotype intensity cluster plots were manually inspected for the 5000 SNVs found to have the lowest *P*-values in a preliminary association test (see below). Where appropriate, genotypes were manually “rescued” using Evoker (version 2.3) (60).

#### German and Estonian data

We removed samples from the German and Estonian cohorts with high missingness (> 2%). SNVs were removed if they had low call rate (< 95%) or deviated from Hardy Weinberg equilibrium (*P* < 0.0001) across both cohorts combined. Triallelic variants, insertions, deletions and one of each pair of duplicated markers were excluded. Rare variant genotypes were called using the zCall algorithm after removing samples with a call rate < 95%. zCall was employed using default settings (59) for the German and Estonian cohorts separately.

#### Michigan data

We removed samples with high missingness (> 2%), and markers with low call rate (< 95%) or that deviated from Hardy Weinberg equilibrium (*P* < 1 × 10^−6^). Additional QC steps and rare variant calling using zCall were performed in Kiel as described above for the German and Estonian datasets.

#### Joint quality control

All four datasets were filtered to exclude variants with call rate below 99% and samples with missingness above 1%. We took forward for subsequent analysis only those SNVs that were present in all four datasets. We excluded SNVs where alleles did not match between datasets or where the minor allele was ambiguous (i.e. symmetrical SNVs with MAF > 0.45 and disagreement between datasets).

A subset of 32 403 independent SNVs was generated by excluding SNVs with MAF < 0.001 and SNVs within 250 kb of a previously-published psoriasis susceptibility locus or in regions of long-range LD as defined by Price *et al.* (61); and by using PLINK to perform LD-pruning (*r*^2^ threshold = 0.2). Relationship inference was performed jointly across all samples based on this independent subset of SNVs, using KING (version 1.4) (62). For pairs of samples found to be related (second degree relative or closer; kinship coefficient > 0.0884), the sample with fewer missing genotypes was retained and the other excluded from further analysis. For each of the four datasets separately, principal component analysis (PCA) was performed based on the SNVs within the independent subset having MAF > 0.01 in that dataset (between 16 307 and 16 629 SNVs). In order to mitigate against population stratification we excluded PCA outliers from all four datasets (defined as samples lying > 6 standard deviations away from the mean for any of the first ten principal components) (Supplementary Material, Fig. S1).

Following an initial round of association testing (described below), genotype intensity cluster plots for all non-MHC variants with single variant association *P*-value < 10^−5^ or included in a gene achieving a *P*-value < 10^−5^ in any aggregation test were manually inspected (and if necessary, removed or manually corrected) in all four datasets using Evoker (60). All analysis was subsequently repeated using these final datasets to give the results presented in this article. Cluster plots have been checked in the final datasets for all variants and genes reported here.

### Linkage disequilibrium

All LD statistics reported in this work derive from 503 samples of European ancestry from 1000 Genomes (phase 3) (63). Estimates of *r*^2^ and *D*’ were calculated using PLINK.

### Proxy markers for established psoriasis susceptibility variants

We curated established genome-wide significant psoriasis susceptibility variants from the literature (5–17,35,64) (Supplementary Material, Table S6). Where established psoriasis variants were not present in our study, we identified tested SNVs within 500 kb with which they are in LD (*r*^2^ > 0.8); of these, we used the SNV in strongest LD as a proxy for the established variant.

### Single marker association testing

We used a linear mixed model (LMM) implemented in EMMAX (27) to test for association of single variants in each of the four studies. In each study population, structure was controlled for using a genetic relatedness matrix derived from the set of 32 403 independent SNVs described above; to avoid confounding due to LD and known psoriasis association, we also estimated genomic inflation using the *P*-values of association for these SNVs. Evaluation of quantile-quantile (QQ) plots indicated that inflation was minimal (Supplementary Material, Fig. S2), with median genomic control (λ_GC_) values ranging from 1.005 to 1.048 across the four studies. We subsequently performed standard-error weighted fixed-effect meta-analysis using METAL (current version) (65) to obtain combined *P*-values. Since EMMAX does not guarantee the accuracy of effect size estimates for binary traits, we estimated odds ratios (ORs) separately. For this we used PLINK (v1.9; www.cog-genomics.org/plink/1.9/; date last accessed August 15, 2017) (66) to perform logistic regression for each study with the first ten principal components as covariates, and the ‘meta’ package (67) in R for meta-analysis. We verified that the *P*-values generated under this method are consistent with our primary results generated by the LMM (Supplementary Material, Fig. S3).

Single variants were only considered significantly associated with psoriasis susceptibility if their direction of effect was consistent across all four studies and *P*-value of association was below the exome-wide significance threshold of 3.0 × 10^−7^ (corresponding to 0.05/167 587 variants tested).

Where significantly associated protein-altering variants were identified in established psoriasis susceptibility loci, we assessed the degree to which each protein-altering variant corresponds to established association signal. This was done by estimating LD between the protein-altering variant and the known associated variant, and where a suitable proxy for the known variant existed in our data, by performing (bidirectional) conditional association testing with the protein-altering variant. Conditional association *P*-values were generated using EMMAX and METAL as above, with the genotype of the SNV to be conditioned on included as a fixed covariate in EMMAX and with the same genetic relatedness matrices as the unconditioned analysis.

### Gene-based association testing

We prepared genotype data for gene-based association testing using EPACTS (v3.2.3; http://genome.sph.umich.edu/wiki/EPACTS; date last accessed August 15, 2017) to annotate variants. We used RAREMETALWORKER (v4.13.5) (68) to generate score statistics and covariance information based on individual markers in each study; population structure was controlled for using a genetic relatedness matrix derived from the set of 32 403 independent SNVs described above. We subsequently used rareMETALS2 (v0.1; http://genome.sph.umich.edu/wiki/RareMETALS2; date last accessed August 15, 2017) to perform combined gene-level meta-analysis, for each gene including all variants annotated as protein-altering (nonsynonymous, stop-gain and essential splice site) and having MAF below a fixed threshold. These combined tests comprised the GRANVIL (69) (burden) test and SKAT (30) (variance component) test, using MAF thresholds of both 0.01 and 0.05. To correct for exome-wide testing, we used a Bonferroni-corrected threshold of 0.05/20,000 = 2.5 × 10^−6^ to classify genes as significantly associated with psoriasis susceptibility. Since RAREMETALWORKER and rareMETALS2 also provide single marker association test results we confirmed that meta-analysis *P*-values and effect sizes generated in this way are consistent with our primary results obtained as described above (Supplementary Material, Fig. S4 and Table S4).

ORs were estimated for gene-based tests by collapsing all included rare variants across each gene into a single genotype, and performing logistic regression in PLINK and meta-analysis using the R ‘meta’ package as for single marker association testing (described above).

Since both genes achieving exome-wide significance fell within established psoriasis susceptibility loci for which exome-wide significant single variants were identified by our earlier analysis, we tested for gene-level association signal that could be attributed to rare variants independently of these known single variants. This was done by repeating the gene-level association tests and conditioning on the associated single variants, using the conditional analysis function implemented in rareMETALS2 (and excluding from the set of variants to be aggregated any associated single variants with sufficiently low MAF to be otherwise included).

We further investigated genes in all established psoriasis susceptibility loci. Our data included rare or low-frequency protein-altering variants in 412 genes located within 250 kb of a previously or newly reported single variant association. We checked these genes for GRANVIL and SKAT test *P*-values below a threshold of 0.05/412 = 1.214 × 10^−4^.

### Variant effect

We predicted variant effects using three *in silico* tools. We consider SIFT (70) scores below 0.05, PolyPhen-2 (71) estimated false-positive rate below 0.05 and scaled CADD (72) scores above 20 to indicate a predicted functional effect. For all variants, scores for all three prediction tools were generated via wANNOVAR (73). All amino acid substitutions described refer to the canonical protein sequence as defined by UniProt (74).

### Exome array coverage

We collated variants included in the original exome array design from the online documentation (http://genome.sph.umich.edu/wiki/Exome_Chip_Design; date last accessed August 15, 2017). Variants subsequently included on each of the genotyping arrays used were obtained from the relevant manufacturer (Illumina or Affymetrix; Supplementary Material, Table S1).

To estimate the coverage of protein-altering variants by the genotyping arrays we downloaded annotated ExAC variants (release 0.3.1) (75). Biallelic SNVs were extracted which included an annotation of moderate or high impact to at least one protein-coding transcript. We further filtered these variants to those with non-zero alternative allele count in 33 370 European samples based on variant calls for at least 10 000 chromosomes. After excluding SNVs on non-autosomal chromosomes and those within the MHC region this resulted in 1 655 908 SNVs, of which 14 123 were common (MAF ≥ 0.05), 9957 were low-frequency (0.01 ≤ MAF < 0.05), 32 029 were rare but not very rare (0.001 ≤ MAF < 0.01). The majority (1 599 799) had MAF below 0.001.

To assess coverage of potential causal variants in established non-MHC psoriasis susceptibility loci, we searched for variants in 1000 Genomes European samples that are in moderate LD (*r*^2^ ≥ 0.2) with a previously reported association, as described above. This resulted in 17 215 SNVs in total. To identify candidate exonic variants we extracted those which included a SIFT, PolyPhen or CADD annotation predicting impaired protein function.

## Supplementary Material

Supplementary Material is available at *HMG* online.

## Supplementary Material

Supplementary Figures and Tables

Supplementary Data
