# Supplementary material for: Exome-wide association study reveals novel psoriasis susceptibility locus at TNFSF15 and rare protective alleles in genes contributing to type I IFN signalling
Source: Hum Mol Genet. 2017 Aug 24;26(21):4301–13. doi: 10.1093/hmg/ddx328 (PMC5886170; doi:10.1093/hmg/ddx328)
Supplement: Supplementary Figures and Tables [file dand_exomewideassociationstudy_hmg_2017_supplementary_ddx328.doc]

Supplementary Information

**Exome-wide association study reveals novel psoriasis susceptibility locus at *TNFSF15* and rare protective alleles in genes contributing to type I IFN signalling**

Nick Dand*, Sören Mucha*, Lam C. Tsoi*, Satveer K. Mahil, Philip E. Stuart, Andreas Arnold, Hansjörg Baurecht, A. David Burden, Kristina Callis Duffin, Vinod Chandran, Charles J. Curtis, Sayantan Das, David Ellinghaus, Eva Ellinghaus, Charlotta Enerback, Tõnu Esko, Dafna D. Gladman, Christopher E. M. Griffiths, Johann E. Gudjonsson, Per Hoffman, Georg Homuth, Ulrike Hüffmeier, Gerald G. Krueger, Matthias Laudes, Sang Hyuck Lee, Wolfgang Lieb, Henry W. Lim, Sabine Löhr, Ulrich Mrowietz, Martina Müller-Nurayid, Markus Nöthen, Annette Peters, Proton Rahman, André Reis, Nick J. Reynolds, Elke Rodriguez, Carsten O. Schmidt, Sarah L. Spain, Konstantin Strauch, Trilokraj Tejasvi, John J. Voorhees, Richard B. Warren, Michael Weichenthal, Stephan Weidinger, Matthew Zawistowski, Rajan P. Nair, Francesca Capon, Catherine H. Smith, Richard C. Trembath, Goncalo R. Abecasis, James T. Elder^†^, Andre Franke^†^, Michael A. Simpson^†^, Jonathan N. Barker^†^

* These authors contributed equally to this work

^†^ These authors jointly supervised this work

**Contents**

Supplementary Figure 1 – Principal component analysis

Supplementary Figure 2 – Single marker Q-Q plots

Supplementary Figure 3 – P-value agreement with PLINK + ‘meta’ analysis

Supplementary Figure 4 – P-value agreement with RAREMETALWORKER + rareMETALS2 analysis

Supplementary Table 1 – Description of datasets

Supplementary Table 2 – Summary of QC process

Supplementary Table 3 – Summary of genotyped variants

Supplementary Table 4 – Effect size agreement with RAREMETALWORKER + rareMETALS2 analysis

Supplementary Table 5 – Full results for all single markers displaying evidence for association

Supplementary Table 6 – Association test results for established psoriasis susceptibility loci

Supplementary Table 7 – Conditional analysis of multiple protein-altering SNVs found to be associated in the same loci

Supplementary Table 8 – Conditional test results for genes implicated by aggregation tests

Supplementary Table 9 – Significant gene-based associations within known psoriasis loci

Supplementary Table 10 – Full single marker association test results for rare protein-altering variants in genes implicated by aggregation tests

Supplementary Table 11 – Summary of coverage for predicted damaging autosomal coding variants in ExAC

Supplementary Table 12 – Genotyping and association test results for potential causal SNVs identified in 1000 Genomes data

Supplementary Note – Summary of evidence that protein-altering variants contribute to psoriasis pathogenesis, by locus

References

**Supplementary Figure 1 – Principal component analysis**

Plots of first two principal components by study, based on principal component analysis of final samples after quality control steps.

**
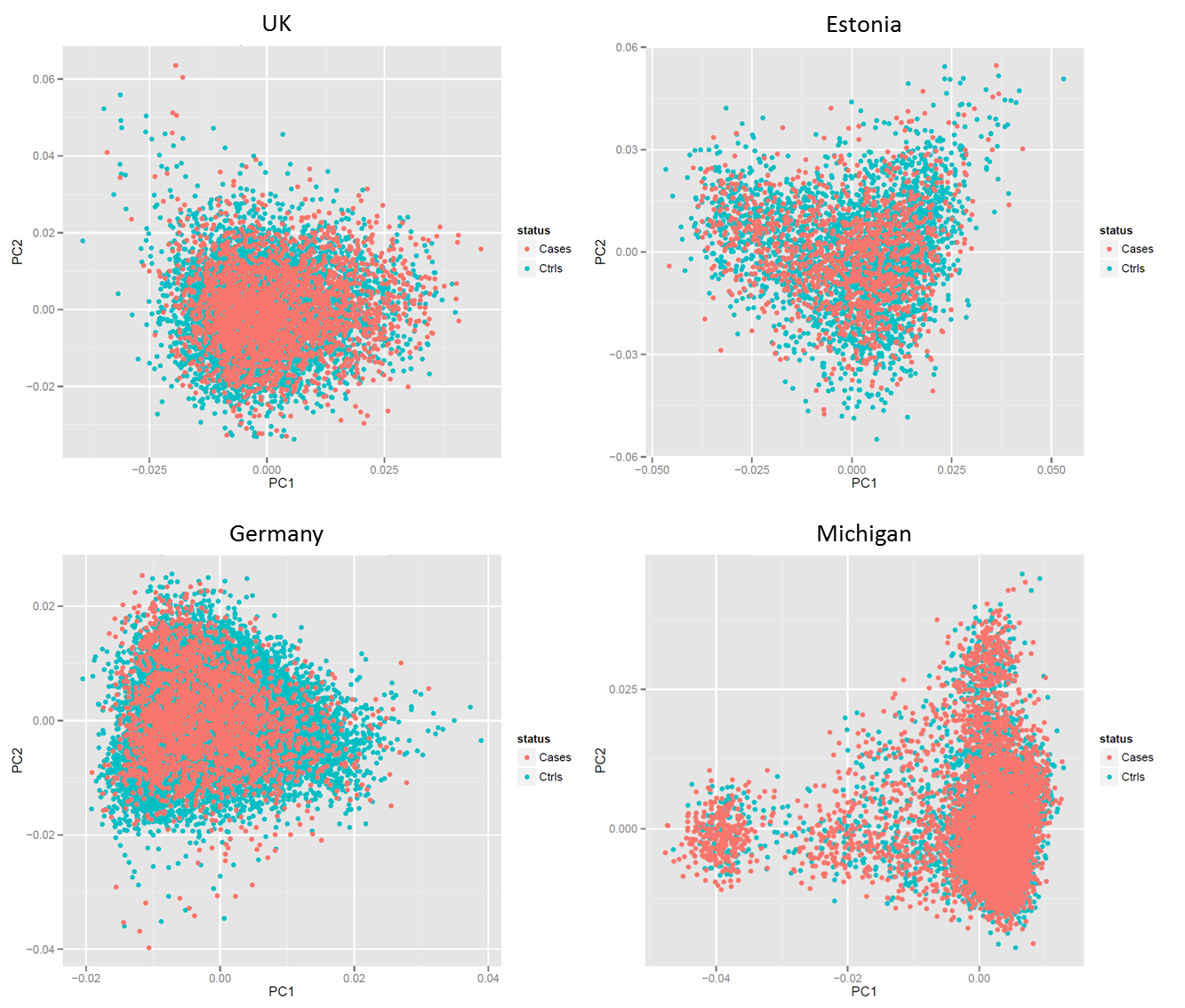
**

**Supplementary Figure 2 – Single marker Q-Q plots**

Plots derived from association p-values for the set of 32,403 independent SNVs described in the main text (Materials and Methods).

**
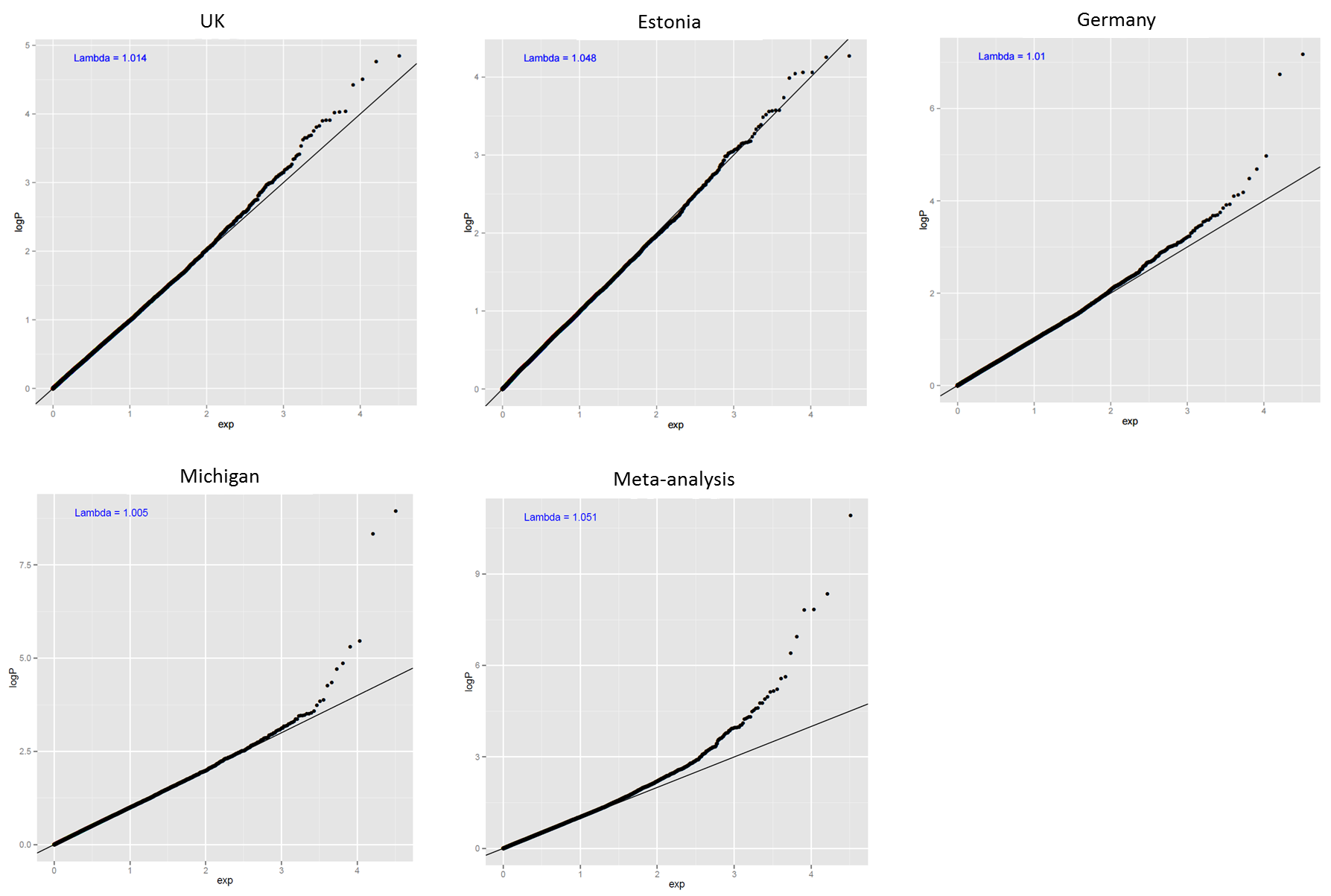
**

**Supplementary Figure 3 – P-value agreement with PLINK + ‘meta’ analysis**

Single variant meta-analysis p-values derived via PLINK logistic regression followed by meta-analysis using the ‘meta’ package in R are consistent with those derived via linear mixed model in EMMAX followed by meta-analysis in METAL. All variants: includes all variants for which a p-value is obtained under both methods; associated variants: those variants achieving exome-wide significance in EMMAX + METAL (*p* < 3.0×10^‑7^); orange dashed line: exome-wide significance threshold.


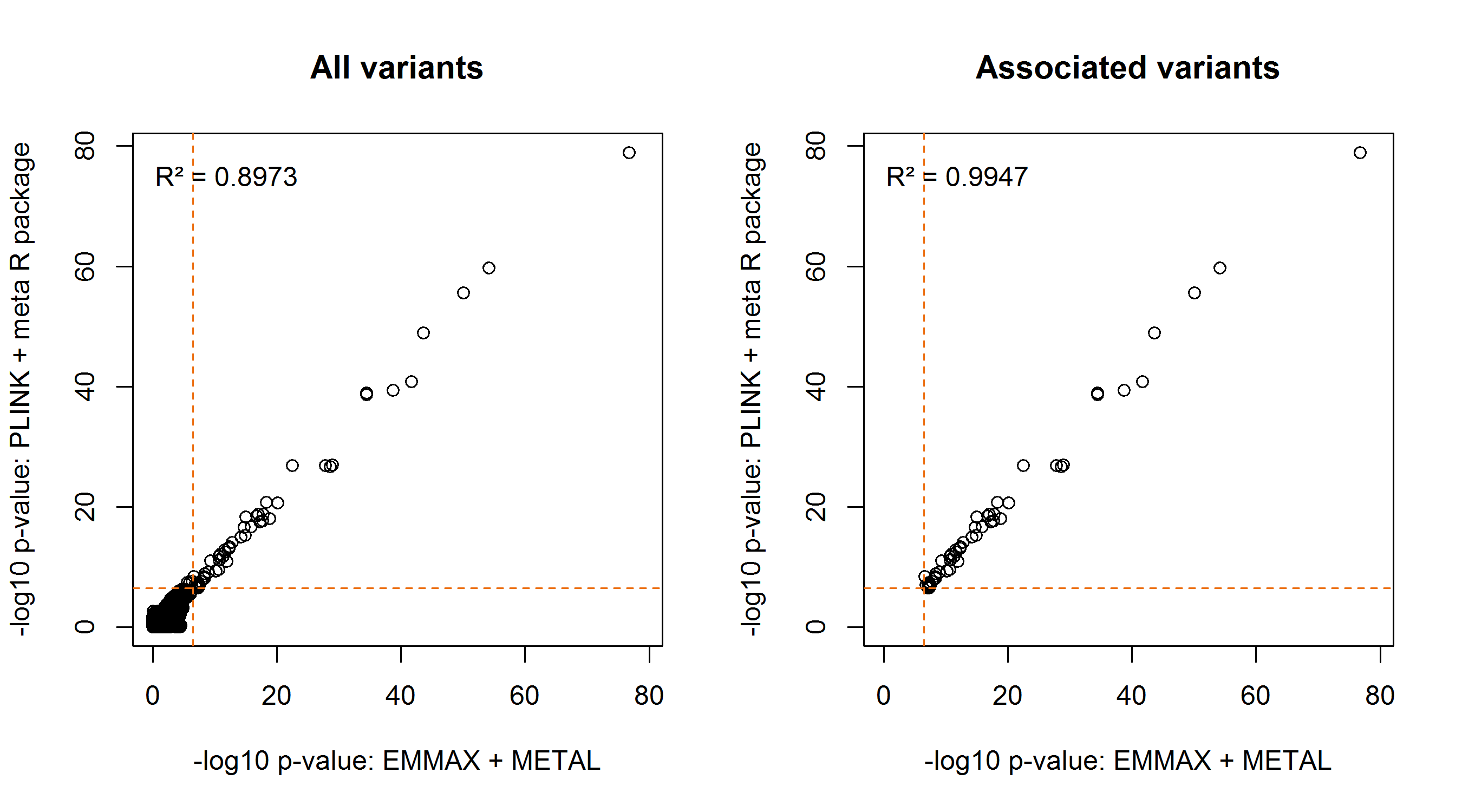


**Supplementary Figure 4 – P-value agreement with RAREMETALWORKER + rareMETALS2 analysis**

Single variant meta-analysis p-values derived via RAREMETALWORKER followed by meta-analysis using rareMETALS2 are consistent with those derived via EMMAX followed by meta-analysis in METAL. Blue dashed line: exome-wide significance threshold (3.0×10^‑7^).


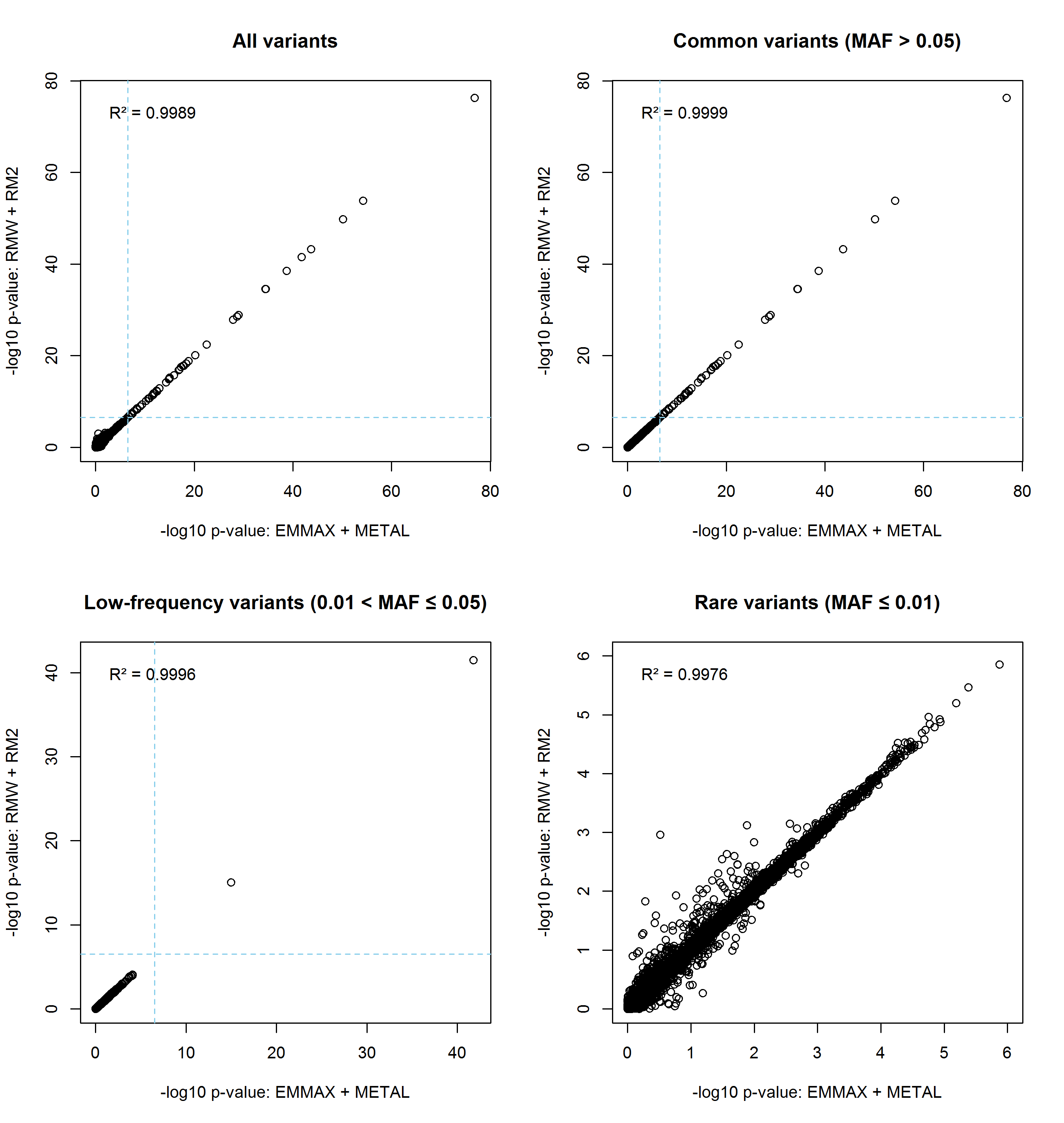


**Supplementary Table 1 – Description of datasets**

QC: quality control; GAPC: Genetic Analysis of Psoriasis Consortium; B-STOP: Biomarkers of Systemic Treatment Outcomes in Psoriasis study.

| **Study** | **Source** | **Number of cases** | **Number of controls** | **Final number of cases after QC** | **Final number of controls after QC** | **Genotyping chip** |
| --- | --- | --- | --- | --- | --- | --- |
| UK | GAPC | 1,971 ^[a]^ | - | 1,593 | - | Illumina HumanExome-12 v1.1 BeadChip |
|  | B-STOP | 960 | - | 838 | - | Illumina HumanOmniExpressExome-8 v1.2 |
|  | 1958 Birth cohort | - | 6,400 | - | 5,892 | Illumina HumanExome-12 v1.0 BeadChip |
|  | **Total** | **2,931** | **6,400** | **2,431** | **5,892** |  |
| Estonia | ExomeChip1.1 cases/controls | 988 | 2,896 | 841 | 2,485 | Illumina HumanExome-12 v1.1 Beadchip |
|  | HumanCore24Exome cases/controls | 471 | 271 | 423 | 247 | Illumina HumanCoreExome-24 v1.0 |
|  | **Total** | **1,459 [**^b]^ | **3,167 [**^c]^ | **1,264** | **2,732** |  |
| Germany | ExomeChip1.0 controls | - | 15,242 | - | 13,958 | Illumina HumanExome-12 v1.0 Beadchip |
|  | ExomeChip1.1 cases | 1,545 | - | 1,288 | - | Illumina HumanExome-12 v1.1 Beadchip |
|  | HumanCore12Exome cases | 1,008 | - | 921 | - | Illumina HumanCoreExome-12 v1.1B |
|  | HumanCore24Exome cases/controls | 375 | 44 | 299 | 15 | Illumina HumanCoreExome-24 v1.0 A |
|  | OmniExpressExome controls | - | 680 | - | 568 | Illumina HumanOmniExpressExome-8 v1.2 A |
|  | **Total** | **2,928** ^[d]^ | **15,966** ^[e]^ | **2,508** | **14,541** |  |
| Michigan | USA | 4,242 | 4,470 | 3,719 | 3,961 | Affymetrix Axiom Psoriasis Michigan SNP Array |
|  | Canada | 1,592 | 1,125 | 1,487 | 1,079 |  |
|  | Sweden | 510 | 490 | 452 | 405 |  |
|  | **Total** | **6,344** ^[f]^ | **6,085** | **5,658** | **5,445** |  |
| **Total** |  | **13,662** | **31,618** | **11,861** | **28,610** |  |

^[a]^ These psoriasis cases were analysed previously in ref. (1).

^[b]^ Includes 1,190 psoriasis cases analysed previously in ref. (1).

^[c]^ Includes 45 controls analysed previously in ref. (1).

^[d]^ Includes 551 psoriasis cases analysed previously in ref (1) and 389 in ref. (2).

^[e]^ Includes 1,565 controls analysed previously in ref. (1) and 547 in ref. (2).

^[f]^ Includes 1,523 psoriasis cases analysed previously in ref. (3) and 2,261 in ref. (4).

**Supplementary Table 2 – Summary of QC process**

QC: quality control; HWE: Hardy-Weinberg equilibrium; MAF: minor allele frequency.

| **Individual studies** | **UK** (Illumina) | **Estonia** (Illumina) | **Germany** (Illumina) | **Michigan** (Affymetrix) |
| --- | --- | --- | --- | --- |
| Number of samples | 9,331 | 4,626 | 18,894 | 12,429 |
| Number of sites genotyped in all samples | 240,052 | 238,312 | 238,134 | 695,187 |
| Criteria for excluding samples/variants during independent QC at each centre | Samples:   - call rate < 0.95 - non-European ancestry - excess heterozygosity (±4 s.d. from the mean) - array signal intensity > 4 s.d. from the mean - relatedness - revised call rate < 0.99 in remaining variants   Variants:   - call rate < 0.99 - HWE p-value < 0.0001 - cluster separation < 0.4 - duplicated variants - tri-allelic variants - cluster plot check for top 5,000 associations in preliminary analysis - revised call rate < 0.99 in remaining samples | Samples:   - call rate < 0.98   Variants:   - variants not typed in German cohort - call rate < 0.95 (combined with German cohort) - HWE p-value < 0.0001 (combined with German cohort) - duplicated variants - tri-allelic variants | Samples:   - call rate < 0.98   Variants:   - variants not typed in Estonian cohort - call rate < 0.95 (combined with Estonian cohort) - HWE p-value < 0.0001 (combined with Estonian cohort) - duplicated variants - tri-allelic variants | Samples:   - call rate < 0.98 - non-European ancestry   Variants:   - call rate < 0.95 - HWE p-value < 1×10^-6^ - insertions/deletions |
| Number of samples after independent QC | 8,323 | 4,026 | 17,054 | 11,150 |
| Number of sites after independent QC | 234,976 | 228,879 | 228,879 | 626,767 |
| *(of which polymorphic)* | *141,524* | *93,841* | *152,304* | *586,547* |
| Criteria for excluding samples/variants during centrally-performed QC | Samples:   - call rate < 0.99 in remaining variants   Variants:   - call rate < 0.99 in remaining samples - insertions/deletions | | | Samples:   - call rate < 0.99 in remaining variants   Variants:   - call rate < 0.99 in remaining samples - variants not typed on Illumina chips |
| Number of samples after QC | 8,323 | 4,026 | 17,054 | 11,147 |
| Number of sites after QC | 234,848 | 228,183 | 228,758 | 172,862 |
| *(of which polymorphic)* | *141,451* | *93,426* | *152,222* | *152,578* |
|  | | | | |
| **Joint QC** | | | | |
| Number of samples in all studies | 40,550 | | | |
| Number of sites passing QC in all studies | 172,440 | | | |
| Criteria for excluding samples/variants during joint QC | Samples:   - one per pair of related samples across all studies (kinship coefficient > 0.0884; sample with fewer missing variants retained) - non-European ancestry (>6 s.d. from the mean for any of PCs 1-10 in per-study PCA)   Variants:   - allele mismatch between studies - ambiguous variants (symmetrical, MAF > 0.45) - cluster plot check for associations in preliminary analysis (*p* < 10^-5^) - HLA region variants - Mitochondrial DNA variants | | | |
| Final number of samples after QC | 40,471 | | | |
| Final number of sites after QC | 167,587 | | | |
| *(of which polymorphic)* | *157,217* | | | |

**Supplementary Table 3 – Summary of genotyped variants**

MAF: minor allele frequency across cases and controls; other start/stop: includes start-gain, start-loss and stop-loss.

| **Class** | **Number of variants** | | | |
| --- | --- | --- | --- | --- |
|  | **Rare**  MAF ≤ 0.01 | **Low-frequency**  0.01 ≤ MAF < 0.05 | **Common**  MAF > 0.05 | **Total** |
| Synonymous | 5,058 | 251 | 600 | 5,909 |
|  | 3.0% | 0.1% | 0.4% | 3.5% |
| Non-synonymous | 130,610 | 5,591 | 8,432 | 144,633 |
|  | 77.9% | 3.3% | 5.0% | 86.3% |
| Stop-gain | 3,301 | 50 | 50 | 3,401 |
|  | 2.0% | 0.0% | 0.0% | 2.0% |
| Other start/stop | 452 | 27 | 31 | 510 |
|  | 0.3% | 0.0% | 0.0% | 0.3% |
| Splice site | 1,322 | 28 | 67 | 1,417 |
|  | 0.8% | 0.0% | 0.0% | 0.8% |
| Non-coding | 3,738 | 507 | 7,472 | 11,717 |
|  | 2.2% | 0.3% | 4.5% | 7.0% |
| **Total** | **144,481** | **6,454** | **16,652** | **167,587** |
|  | 86.2% | 3.9% | 9.9% | 100% |

**Supplementary Table 4 – Effect size agreement with RAREMETALWORKER + rareMETALS2 analysis**

The single marker effect size estimates generated by RAREMETALWORKER followed by meta-analysis using rareMETALS2 are strongly correlated with the estimates from our primary analysis (generated by logistic regression in PLINK followed by meta-analysis using the ‘meta’ R package). Effect size correlation: Pearson correlation between rareMETALS2 beta coefficient and ‘meta’ log(odds ratio); exome-wide significant: variants that we report to be exome-wide significant based on our primary analysis (*p* < 3.0×10^‑7^ using EMMAX and METAL).

| **Variants** | **Effect size**  **correlation** |
| --- | --- |
| Common  MAF > 0.05 | 0.9615 |
| Low-frequency  0.01 ≤ MAF < 0.05 | 0.9475 |
| Rare  MAF ≤ 0.01 | 0.8357 |
| All | 0.8381 |
| Exome-wide significant | 0.9972 |

**Supplementary Table 5 – Full results for all single markers displaying evidence for association**

See Supplementary Excel workbook.

**Supplementary Table 6 – Association test results for established psoriasis susceptibility loci**

See Supplementary Excel workbook.

**Supplementary Table 7 – Conditional analysis of multiple protein-altering SNVs found to be associated in the same loci**

| **Locus** | **SNV 1** | **SNV2** | **Meta-analysis p-value** | |
| --- | --- | --- | --- | --- |
|  |  |  | **SNV 1 conditioned on SNV 2** | **SNV 2 conditioned on SNV 1** |
| 1q21.3 | rs1332500 | rs873775 | 0.2524 | 0.2773 |
| 2q24.2 | rs1990760 | rs35667974 | 0.9619 | 2.58×10^-11^ |
| 5q15 | rs27044 | rs30187 | 0.9881 | 0.2807 |
| 6q21 | rs33980500 | rs13190932 | 0.0816 | 0.1115 |
|  | rs33980500 | rs458017 | 4.04×10^-7^ | 0.9896 |
|  | rs13190932 | rs458017 | 2.41×10^-7^ | 0.2538 |
| 19p13.2 | rs34536443 | rs2304256 | 2.63×10^-29^ | 0.0559 |
|  | rs34536443 | rs12720356 | 1.13×10^-45^ | 4.37×10^-6^ |
|  | rs34536443 | rs1051738 | 2.42×10^-36^ | 0.6174 |
|  | rs2304256 | rs12720356 | 0.1063 | 0.0032 |
|  | rs2304256 | rs1051738 | 2.04×10^-4^ | 0.0106 |
|  | rs12720356 | rs1051738 | 1.21×10^-5^ | 2.41×10^-9^ |

**Supplementary Table 8 – Conditional test results for genes implicated by aggregation tests**

MAF = minor allele frequency; *n*_SNVs_ = number of SNVs included in test (this may vary between tests since SNVs to be conditioned on which are sufficiently rare are not included in the test statistic); cMAF = cumulative minor allele frequency of SNVs included in test. Exome-wide significant p-values (*p* < 2.5×10^‑6^) are indicated in bold.

| **Locus** | **Gene** | **SNVs conditioned on** | **MAF < 0.01** | | | | **MAF < 0.05** | | | |
| --- | --- | --- | --- | --- | --- | --- | --- | --- | --- | --- |
|  |  |  | ***p*_burden_** | ***p*_SKAT_** | ***n*_SNVs_** | **cMAF** | ***p*_burden_** | ***p*_SKAT_** | ***n*_SNVs_** | **cMAF** |
| 2q24.2 | *IFIH1* | rs35667974 | **1.44×10^-7^** | 3.49×10^-5^ | 24 | 0.0261 | **1.44×10^-7^** | 3.49×10^-5^ | 24 | 0.0261 |
|  |  | rs1990760 | **1.55×10^-8^** | 3.64×10^-6^ | 24 | 0.0261 | **1.24×10^-17^** | **8.48×10^-17^** | 25 | 0.0461 |
|  |  | rs35667974 and rs1990760 | **1.36×10^-8^** | 4.46×10^-6^ | 24 | 0.0261 | **1.36×10^-8^** | 4.46×10^-6^ | 24 | 0.0261 |
| 19p13.2 | *TYK2* | rs34536443 | 1.39×10^-4^ | 6.85×10^-5^ | 17 | 0.0115 | 4.33×10^-5^ | 1.07×10^-4^ | 18 | 0.0239 |
|  |  | rs2304256 | 0.0232 | 0.0151 | 17 | 0.0115 | **3.70×10^-28^** | **1.67×10^-26^** | 19 | 0.0675 |
|  |  | rs34536443 | 3.37×10^-4^ | 1.38×10^-4^ | 17 | 0.0115 | **6.01×10^-43^** | **3.52×10^-44^** | 19 | 0.0675 |
|  |  | rs34536443, rs2304256 and rs12720356 | 1.45×10^-4^ | 7.21×10^-5^ | 17 | 0.0115 | 1.85×10^-5^ | 6.53×10^-5^ | 18 | 0.0239 |

**Supplementary Table 9 – Significant gene-based associations within known psoriasis loci**

MAF = minor allele frequency; OR = odds ratio estimated by collapsing test; nSNVs = number of SNVs included in test (this may vary between unconditioned and conditional analysis since SNVs to be conditioned on which are sufficiently rare are not included in the test statistic for the conditional test); cMAF = cumulative minor allele frequency of SNVs included in test. P-values which are significant after Bonferroni correction (*p*_gene_ < 1.613×10^‑4^, based on 310 tested genes within known psoriasis susceptibility loci) are indicated in bold.

| **Locus** | **Gene** | **SNVs conditioned on** | **MAF < 0.01** | | | | | **MAF < 0.05** | | | | |
| --- | --- | --- | --- | --- | --- | --- | --- | --- | --- | --- | --- | --- |
|  |  |  | ***p*_burden_** | **OR (95% CI)** | ***p*_SKAT_** | ***n*_SNVs_** | **cMAF** | ***p*_burden_** | **OR (95% CI)** | ***p*_SKAT_** | ***n*_SNVs_** | **cMAF** |
| ***Unconditioned analysis*** | | | | | | | | | | | | |
| 1p31.3 | *IL23R* | - | **4.13×10^-5^** | 0.682  (0.573 - 0.813) | 3.52×10^-4^ | 5 | 0.0110 | **7.98×10^-6^** | 0.750  (0.668 - 0.842) | 4.50×10^-4^ | 6 | 0.0242 |
| 2q24.2 | *IFIH1* | - | **2.53×10^-7^** | 0.707  (0.626 - 0.799) | **6.02×10^-5^** | 24 | 0.0261 | **1.84×10^-19^** | 0.620  (0.564 - 0.682) | **1.19×10^-20^** | 25 | 0.0461 |
| 6q23.3 | *TNFAIP3* | - | 0.6861 | 0.918  (0.738 - 1.143) | 0.3893 | 9 | 0.0058 | 2.06×10^-4^ | 0.837  (0.765 - 0.916) | **1.16×10^-4^** | 10 | 0.0362 |
| 9p21.1 | *DDX58* | - | **1.49×10^-4^** | 0.728  (0.616 - 0.860) | **1.41×10^-4^** | 9 | 0.0117 | **3.01×10^-5^** | 0.792  (0.710 - 0.884) | **7.82×10^-5^** | 10 | 0.0266 |
| 12q13.3 | *STAT2* | - | 0.2047 | 0.834  (0.458 - 1.520) | 0.2409 | 6 | 0.0010 | **3.80×10^-5^** | 0.698  (0.595 - 0.820) | **9.48×10^-5^** | 7 | 0.0126 |
| 19p13.2 | *TYK2* | - | 6.17×10^-4^ | 0.744  (0.626 - 0.885) | 2.82×10^-4^ | 17 | 0.0115 | **1.47×10^-39^** | 0.593  (0.549 - 0.641) | **6.34×10^-41^** | 19 | 0.0675 |
| ***Conditional analysis*** | | | | | | | | | | | | |
| 1p31.3 | *IL23R* | rs11209026 | **1.56×10^-5^** | 0.670  (0.563 - 0.799) | **1.44×10^-4^** | 5 | 0.0110 | **1.60×10^-6^** | 0.737  (0.656 - 0.827) | **1.36×10^-4^** | 6 | 0.0242 |
| 2q24.2 | *IFIH1* | rs35667974 and rs1990760 | **1.36×10^-8^** | 0.687  (0.607 - 0.776) | **4.46×10^-6^** | 24 | 0.0261 | **1.36×10^-8^** | 0.687  (0.607 - 0.776) | **4.46×10^-6^** | 24 | 0.0261 |
| 6q23.3 | *TNFAIP3* | rs610604 | 0.4449 | 0.878  (0.705 - 1.093) | 0.2117 | 9 | 0.0058 | 2.22×10^-3^ | 0.862  (0.787 - 0.944) | 2.36×10^-3^ | 10 | 0.0362 |
| 9p21.1 | *DDX58* | rs657454 | **2.37×10^-5^** | 0.702  (0.593 - 0.831) | **1.74×10^-5^** | 9 | 0.0117 | **3.15×10^-5^** | 0.793  (0.711 - 0.884) | **3.07×10^-5^** | 10 | 0.0266 |
| 12q13.3 | *STAT2* | rs2066808 | 0.5601 | 0.979  (0.536 - 1.790) | 0.7150 | 6 | 0.0010 | **2.48×10^-5^** | 0.692  (0.589 - 0.812) | **2.34×10^-5^** | 7 | 0.0126 |
| 19p13.2 | *TYK2* | rs34536443, rs2304256 and rs12720356 | **1.45×10^-4^** | 0.728  (0.611 - 0.868) | **7.21×10^-5^** | 17 | 0.0115 | **1.85×10^-5^** | 0.790  (0.701 - 0.890) | **6.53×10^-5^** | 18 | 0.0239 |

**Supplementary Table 10 – Full single marker association test results for rare protein-altering variants in genes implicated by aggregation tests**

See Supplementary Excel workbook.

**Supplementary Table 11 – Summary of coverage for predicted damaging autosomal coding variants in ExAC**

Includes all biallelic autosomal non-MHC SNVs in ExAC having non-zero alternative allele count in non-Finnish Europeans based on observations for ≥10,000 chromosomes, and for which at least one annotation indicates moderate or high impact to a protein-coding transcript. MAF = minor allele frequency.

| **ExAC variants** | **Counts by MAF range** | | | | | |
| --- | --- | --- | --- | --- | --- | --- |
|  | < 0.0001 | 0.0001 - 0.001 | 0.001 - 0.01 | 0.01 - 0.05 | ≥ 0.05 | All |
| Total number | 1,455,568 | 144,231 | 32,029 | 9,957 | 14,123 | 1,655,908 |
| In Exome chip design | 53,967 | 67,453 | 26,946 | 8,827 | 12,903 | 170,096 |
|  | 3.7% | 46.8% | 84.1% | 88.7% | 91.4% | 10.3% |
| On Illumina arrays | 45,756 | 54,952 | 21,033 | 6,618 | 9,260 | 137,619 |
|  | 3.1% | 38.1% | 65.7% | 66.5% | 65.6% | 8.3% |
| On Affymetrix array | 55,172 | 53,711 | 21,678 | 7,514 | 10,936 | 149,011 |
|  | 3.8% | 37.2% | 67.7% | 75.5% | 77.4% | 9.0% |
| On both array types | 35,017 | 43,425 | 17,683 | 5,980 | 8,636 | 110,741 |
|  | 2.4% | 30.1% | 55.2% | 60.1% | 61.1% | 6.7% |
| Passed final QC | 33,329 | 41,405 | 16,114 | 5,025 | 7,072 | 102,945 |
|  | 2.3% | 28.7% | 50.3% | 50.5% | 50.1% | 6.2% |

**Supplementary Table 12 – Genotyping and association test results for potential causal SNVs identified in 1000 Genomes data**

See Supplementary Excel workbook.

**Supplementary Note – Summary of evidence that protein-altering variants contribute to psoriasis pathogenesis, by locus**

**1p31.3 –** In *IL23R*, which encodes the interleukin (IL)-23 receptor, we identified a single significantly associated missense SNV, the same potential causal variant suggested by Tsoi *et al.* (1). The variant rs11209026 (*p* = 2.00×10^‑18^; OR = 0.72) causes the substitution p.R381Q which has previously been shown to inhibit Th17 cell effector function, either via defective signal transduction (5) or by disrupting normal splicing (6). It is therefore likely that this SNV underlies the protective genetic associations observed at this locus. In support of this we found that the established (intergenic) susceptibility variant rs9988642 is not associated with psoriasis when conditioning on rs11209026 (*p*_conditional_ = 0.3622 based on a proxy SNV; Supplementary Table 5).

**1q21.3 –** Psoriasis risk at 1q21.3 is thought to be conferred by impaired skin barrier function resulting from mutations affecting the epidermal differentiation complex, specifically the late cornified envelope group 3 (*LCE3*) genes (1, 7). We found two associated SNVs in *C1orf68* (rs1332500: *p* = 1.07×10^‑12^, OR = 1.12; rs873775: *p* = 1.11×10^‑12^, OR = 1.12), which encodes a highly skin-specific protein (8). However, *C1orf68* maps ~100 kb from the closest *LCE3* genes (9) and both associations can be accounted for by the established signal at rs4112788 (10) (Supplementary Table 5). The most likely pathogenic mechanism at this locus therefore remains the well-known 32-kb deletion spanning *LCE3B* and *LCE3C*, which is tagged by rs4112788 and within which copy number is correlated with *LCE3B/C* RNA levels in both normal and lesional psoriatic skin (4, 10).

**2q24.2 –** We found two significantly associated single variants in *IFIH1* as well as independent evidence for association of rare functional alleles. For further detail see the Discussion in the main text.

**5q15 -** We detected two significantly associated missense SNVs in *ERAP1*, an established psoriasis susceptibility gene that encodes a peptidase involved in processing antigens for presentation by MHC class I molecules, and which has been reported to exhibit genetic interaction with the most strongly-associated psoriasis susceptibility locus, HLA-C (1, 4, 11, 12). The SNV rs27044 (*p* = 1.28×10^‑13^; OR = 0.87) was proposed as a putative causal variant at this locus (1), but we also observe that rs30187 (*p* = 2.19×10^‑11^; OR = 0.89) can explain the association signal (Supplementary Table 5). Unlike rs27044, the latter SNV is predicted to be deleterious by both PolyPhen-2 and CADD (Supplementary Table 5).

**5q31.1** – In *IL13* we identified a single significantly associated missense SNV (rs20541: *p* = 3.59×10^‑13^, OR = 1.170), the same potential causal variant suggested by Tsoi et al. (1).

**6q21 –** The most significantly associated SNV at this locus was rs33980500 (*p* = 1.92×10^‑39^; OR = 1.45) in *TRAF3IP2*, a gene that encodes the signaling adaptor protein Act1 and provides a potential link between IL-17-mediated adaptive immune responses and innate NF-κB signaling (13). The minor (T) allele has been demonstrated to confer increased psoriasis risk (1, 2), associated with impaired binding of Act1 to TRAF6 (13). LD with this variant seems likely to explain the associations observed at rs13190932 in *TRAF3IP2* and rs458017 in *REV3L* (Supplementary Table 7), neither of which is predicted to be deleterious by more than one prediction tool (Supplementary Table 5).

**12q13.3** – In *STAT2* we identified a single significantly associated missense SNV (rs2066807: *p* = 1.56×10^‑17^, OR = 0.729), the same potential causal variant suggested by Tsoi et al. (1).

**16p11.2 –** Previously, *FBXL19* was suggested to be the most promising candidate psoriasis gene at this susceptibility locus, due to its putative role in inhibition of NF-κB signaling (14). However, this is a highly gene-dense region manifesting in strong regional LD. Within this region, we found a missense variant in *HSD3B7* (rs9938550: *p* = 4.74×10^‑13^; OR = 0.88), drawing our attention because *Hsd3b7* mutations lead to a scaly skin phenotype in mice (15). However, the SNV rs9938550 is not predicted to be pathogenic and is in strong LD with the *FBXL19* intronic variant rs10782001 (meta-analysis p-value 2.01×10^‑12^; OR = 1.13; *r*^2^ = 0.972). Further studies focusing on the effect of genetic variation on regulatory signals will likely be required to identify causal genetic signals in this region.

**19p13.2 –** We found three significantly associated single variants in *TYK2* as well as independent evidence for association of rare functional alleles. For further detail see the Discussion in the main text. In the same locus we detected a protective association at a missense variant in the phosphodiesterase (PDE) gene *PDE4A* (rs1051738; *p* = 2.02×10^-7^; OR = 0.88). Conditional analysis indicates that this corresponds to the same signal as the *TYK2* variant rs34536443 (*p*_conditional_ = 0.6174). However, we note with interest the pro-inflammatory role played by PDE4 enzymes in keratinocytes and the efficacy of PDE4-inhibitors as a treatment for psoriasis (16, 17). These results highlight the complex patterns of association with psoriasis susceptibility within the 19p13.2 locus and suggest that further detailed investigation is necessary to implicate specific amino acid substitutions in the disease process.

**19q13.33** – At this recently reported susceptibility locus (18) we identified one significantly associated missense variant: rs602662 in the gene *FUT2* (*p* = 3.29×10^‑8^; OR = 1.09). This gene encodes α-(1,2) fucosyltransferase, a constituent enzyme of the Lewis antigen system expressed in secretory epithelial cells (19). It is central to determining an individual’s secretor status (20), which has been associated with protection from and susceptibility to certain viral, bacterial and fungal infections (21-23). The SNV rs602662 causes the substitution p.G258S at an evolutionarily conserved residue (24); it has been associated with Crohn’s disease (25) and primary sclerosing cholangitis (24), yet is not thought to be sufficient to generate inactive FUT2 and cause non-secretor status (26). Conversely, the common truncating SNV rs601338 (p.W154X) was not tested in this study but is in strong LD with rs602662 (*r*^2^ = 0.897) and has been shown to inactivate the fucosyltransferase enzyme (19). Therefore, further investigation is required to determine which mutations at the *FUT2* locus contribute directly to psoriasis pathogenesis.

**20q13.13 –** We found an association at rs4647958 (*p* = 9.21×10^‑10^; OR = 1.16), which causes a valine-to-alanine substitution at position 118 in SNAI1, a transcription factor that plays a key role in epithelial-mesenchymal transition (27). However, this conservative substitution is not predicted to be damaging, and segregates perfectly in 1000 Genomes European samples with the previously reported intronic SNV rs1056198 in *RNF114* (*D*’ = 1, although *r*^2^ = 0.070 due to the difference in MAFs). *RNF114* is thought to influence psoriasis risk through its regulatory role in the innate antiviral response instigated by RIG-I and MDA5 (28). Further studies focusing on the effect of genetic variation on regulatory signals will likely be required to identify causal genetic signals in this region.

**References**

1 Tsoi, L.C., Spain, S.L., Knight, J., Ellinghaus, E., Stuart, P.E., Capon, F., Ding, J., Li, Y., Tejasvi, T., Gudjonsson, J.E. *et al.* (2012) Identification of 15 new psoriasis susceptibility loci highlights the role of innate immunity. *Nat. Genet.*, **44**, 1341-1348.

2 Ellinghaus, E., Ellinghaus, D., Stuart, P.E., Nair, R.P., Debrus, S., Raelson, J.V., Belouchi, M., Fournier, H., Reinhard, C., Ding, J. *et al.* (2010) Genome-wide association study identifies a psoriasis susceptibility locus at TRAF3IP2. *Nat. Genet.*, **42**, 991-995.

3 Nair, R.P., Duffin, K.C., Helms, C., Ding, J., Stuart, P.E., Goldgar, D., Gudjonsson, J.E., Li, Y., Tejasvi, T., Feng, B.J. *et al.* (2009) Genome-wide scan reveals association of psoriasis with IL-23 and NF-kappaB pathways. *Nat. Genet.*, **41**, 199-204.

4 Stuart, P.E., Nair, R.P., Tsoi, L.C., Tejasvi, T., Das, S., Kang, H.M., Ellinghaus, E., Chandran, V., Callis-Duffin, K., Ike, R. *et al.* (2015) Genome-wide Association Analysis of Psoriatic Arthritis and Cutaneous Psoriasis Reveals Differences in Their Genetic Architecture. *Am. J. Hum. Genet.*, **97**, 816-836.

5 Di Meglio, P., Di Cesare, A., Laggner, U., Chu, C.C., Napolitano, L., Villanova, F., Tosi, I., Capon, F., Trembath, R.C., Peris, K. *et al.* (2011) The IL23R R381Q gene variant protects against immune-mediated diseases by impairing IL-23-induced Th17 effector response in humans. *PLOS ONE*, **6**, e17160.

6 Yu, R.Y., Brazaitis, J. and Gallagher, G. (2015) The human IL-23 receptor rs11209026 A allele promotes the expression of a soluble IL-23R-encoding mRNA species. *J. Immunol.*, **194**, 1062-1068.

7 Bergboer, J.G., Tjabringa, G.S., Kamsteeg, M., van Vlijmen-Willems, I.M., Rodijk-Olthuis, D., Jansen, P.A., Thuret, J.Y., Narita, M., Ishida-Yamamoto, A., Zeeuwen, P.L. *et al.* (2011) Psoriasis risk genes of the late cornified envelope-3 group are distinctly expressed compared with genes of other LCE groups. *Am. J. Pathol.*, **178**, 1470-1477.

8 Edqvist, P.H., Fagerberg, L., Hallstrom, B.M., Danielsson, A., Edlund, K., Uhlen, M. and Ponten, F. (2015) Expression of human skin-specific genes defined by transcriptomics and antibody-based profiling. *J. Histochem. Cytochem.*, **63**, 129-141.

9 Zhao, X.P. and Elder, J.T. (1997) Positional cloning of novel skin-specific genes from the human epidermal differentiation complex. *Genomics*, **45**, 250-258.

10 de Cid, R., Riveira-Munoz, E., Zeeuwen, P.L., Robarge, J., Liao, W., Dannhauser, E.N., Giardina, E., Stuart, P.E., Nair, R., Helms, C. *et al.* (2009) Deletion of the late cornified envelope LCE3B and LCE3C genes as a susceptibility factor for psoriasis. *Nat. Genet.*, **41**, 211-215.

11 Strange, A., Capon, F., Spencer, C.C., Knight, J., Weale, M.E., Allen, M.H., Barton, A., Band, G., Bellenguez, C., Bergboer, J.G. *et al.* (2010) A genome-wide association study identifies new psoriasis susceptibility loci and an interaction between HLA-C and ERAP1. *Nat. Genet.*, **42**, 985-990.

12 Zheng, H.F., Zuo, X.B., Lu, W.S., Li, Y., Cheng, H., Zhu, K.J., Yin, X.Y., Zhang, C., Ren, Y.Q., Wang, W.J. *et al.* (2011) Variants in MHC, LCE and IL12B have epistatic effects on psoriasis risk in Chinese population. *J. Dermatol. Sci.*, **61**, 124-128.

13 Hüffmeier, U., Uebe, S., Ekici, A.B., Bowes, J., Giardina, E., Korendowych, E., Juneblad, K., Apel, M., McManus, R., Ho, P. *et al.* (2010) Common variants at TRAF3IP2 are associated with susceptibility to psoriatic arthritis and psoriasis. *Nat. Genet.*, **42**, 996-999.

14 Stuart, P.E., Nair, R.P., Ellinghaus, E., Ding, J., Tejasvi, T., Gudjonsson, J.E., Li, Y., Weidinger, S., Eberlein, B., Gieger, C. *et al.* (2010) Genome-wide association analysis identifies three psoriasis susceptibility loci. *Nat. Genet.*, **42**, 1000-1004.

15 Shea, H.C., Head, D.D., Setchell, K.D. and Russell, D.W. (2007) Analysis of HSD3B7 knockout mice reveals that a 3alpha-hydroxyl stereochemistry is required for bile acid function. *Proc. Natl. Acad. Sci. U. S. A.*, **104**, 11526-11533.

16 Schafer, P. (2012) Apremilast mechanism of action and application to psoriasis and psoriatic arthritis. *Biochem. Pharmacol.*, **83**, 1583-1590.

17 Mazur, M., Karczewski, J., Lodyga, M., Zaba, R. and Adamski, Z. (2015) Inhibitors of phosphodiesterase 4 (PDE 4): A new therapeutic option in the treatment of psoriasis vulgaris and psoriatic arthritis. *J Dermatolog Treat*, **26**, 326-328.

18 Tsoi, L.C., Stuart, P.E., Tian, C., Gudjonsson, J.E., Das, S., Zawistowski, M., Ellinghaus, E., Barker, J.N., Chandran, V., Dand, N. *et al.* (2017) Large scale meta-analysis characterizes genetic architecture for common psoriasis associated variants. *Nat. Commun.*, **8**, 15382.

19 Kelly, R.J., Rouquier, S., Giorgi, D., Lennon, G.G. and Lowe, J.B. (1995) Sequence and expression of a candidate for the human Secretor blood group alpha(1,2)fucosyltransferase gene (FUT2). Homozygosity for an enzyme-inactivating nonsense mutation commonly correlates with the non-secretor phenotype. *J. Biol. Chem.*, **270**, 4640-4649.

20 Serpa, J., Mendes, N., Reis, C.A., Santos Silva, L.F., Almeida, R., Le Pendu, J. and David, L. (2004) Two new FUT2 (fucosyltransferase 2 gene) missense polymorphisms, 739G-->A and 839T-->C, are partly responsible for non-secretor status in a Caucasian population from Northern Portugal. *Biochem. J.*, **383**, 469-474.

21 Carlsson, B., Kindberg, E., Buesa, J., Rydell, G.E., Lidon, M.F., Montava, R., Abu Mallouh, R., Grahn, A., Rodriguez-Diaz, J., Bellido, J. *et al.* (2009) The G428A nonsense mutation in FUT2 provides strong but not absolute protection against symptomatic GII.4 Norovirus infection. *PLOS ONE*, **4**, e5593.

22 Ikehara, Y., Nishihara, S., Yasutomi, H., Kitamura, T., Matsuo, K., Shimizu, N., Inada, K., Kodera, Y., Yamamura, Y., Narimatsu, H. *et al.* (2001) Polymorphisms of two fucosyltransferase genes (Lewis and Secretor genes) involving type I Lewis antigens are associated with the presence of anti-Helicobacter pylori IgG antibody. *Cancer Epidemiol. Biomarkers Prev.*, **10**, 971-977.

23 Thom, S.M., Blackwell, C.C., MacCallum, C.J., Weir, D.M., Brettle, R.P., Kinane, D.F. and Wray, D. (1989) Non-secretion of blood group antigens and susceptibility to infection by Candida species. *FEMS Microbiol. Immunol.*, **1**, 401-405.

24 Folseraas, T., Melum, E., Rausch, P., Juran, B.D., Ellinghaus, E., Shiryaev, A., Laerdahl, J.K., Ellinghaus, D., Schramm, C., Weismuller, T.J. *et al.* (2012) Extended analysis of a genome-wide association study in primary sclerosing cholangitis detects multiple novel risk loci. *J. Hepatol.*, **57**, 366-375.

25 McGovern, D.P., Jones, M.R., Taylor, K.D., Marciante, K., Yan, X., Dubinsky, M., Ippoliti, A., Vasiliauskas, E., Berel, D., Derkowski, C. *et al.* (2010) Fucosyltransferase 2 (FUT2) non-secretor status is associated with Crohn's disease. *Hum. Mol. Genet.*, **19**, 3468-3476.

26 Silva, L.M., Carvalho, A.S., Guillon, P., Seixas, S., Azevedo, M., Almeida, R., Ruvoen-Clouet, N., Reis, C.A., Le Pendu, J., Rocha, J. *et al.* (2010) Infection-associated FUT2 (Fucosyltransferase 2) genetic variation and impact on functionality assessed by in vivo studies. *Glycoconj. J.*, **27**, 61-68.

27 Kaufhold, S. and Bonavida, B. (2014) Central role of Snail1 in the regulation of EMT and resistance in cancer: a target for therapeutic intervention. *J. Exp. Clin. Cancer Res.*, **33**, 62.

28 Bijlmakers, M.J., Kanneganti, S.K., Barker, J.N., Trembath, R.C. and Capon, F. (2011) Functional analysis of the RNF114 psoriasis susceptibility gene implicates innate immune responses to double-stranded RNA in disease pathogenesis. *Hum. Mol. Genet.*, **20**, 3129-3137.
